# Biological direct-shortcut deep residual learning for sparse visual processing

**DOI:** 10.1038/s41598-024-62756-y

**Published:** 2024-06-18

**Authors:** Mohammad Mahdi Jahani Yekta

**Affiliations:** Department of Computer Science, Stanford University, 353 Jane Stanford Way, Stanford, CA 94305 USA

**Keywords:** Sparse visual processing, Primary visual cortex, Synaptic weight matrices, Identity mapping, Deep residual learning, Direct-shortcuts, Computational biology and bioinformatics, Engineering, Mathematics and computing

## Abstract

We show, based on the following three grounds, that the primary visual cortex (V1) is a biological direct-shortcut deep residual learning neural network (ResNet) for sparse visual processing: (1) We first highlight that Gabor-like sets of basis functions, which are similar to the receptive fields of simple cells in the primary visual cortex (V1), are excellent candidates for sparse representation of natural images; i.e., images from the natural world, affirming the brain to be optimized for this. (2) We then prove that the intra-layer synaptic weight matrices of this region can be reasonably first-order approximated by identity mappings, and are thus sparse themselves. (3) Finally, we point out that intra-layer weight matrices having identity mapping as their initial approximation, irrespective of this approximation being also a reasonable first-order one or not, resemble the building blocks of direct-shortcut digital ResNets, which completes the grounds. This biological ResNet interconnects the sparsity of the final representation of the image to that of its intra-layer weights. Further exploration of this ResNet, and understanding the joint effects of its architecture and learning rules, e.g. on its inductive bias, could lead to major advancements in the area of bio-inspired digital ResNets. One immediate line of research in this context, for instance, is to study the impact of forcing the direct-shortcuts to be good first-order approximations of each building block. For this, along with the ${{\ell}}_{1}$-minimization posed on the basis function coefficients the ResNet finally provides at its output, another parallel one could e.g. also be posed on the weights of its residual layers.

## Introduction

Sparse signal representation refers to decomposition of signals using just a few basis functions, also known as kernels or atoms, and has been the topic of a wide spectrum of researches. It has application in many areas such as 1. image denoising and restoration, 2. signal sampling and recovery, 3. source localization with sensor arrays, 4. sound source separation, 5. biomedical imaging, signal analysis, and extraction, 6. independent component analysis (ICA), 7. synthetic aperture radar (SAR) image formation, 8. sub-band adaptive filtering, 9. unsupervised analysis of signals, 10. minimum rank approximation, 11. 3D dense medium simulations, 12. signal classification, 13. facial expression recognition, and 14. blind spectral unmixing. Many works have also been done on the peripheral topics related to it, such as analysis of noise sensitivity of sparse representations, and using of a priori information for such approximations.

Considering the approximately 80% of the information perceived by humans being of visual type, image signals and their sparse representations have always received particular attention. We may refer in this context e.g. to deep view synthesis from sparse photometric images, fine-grained image recognition, and deep learning opto-acoustic tomography.

In this paper we show that the primary visual cortex (V1) is a biological direct-shortcut deep residual learning neural network (ResNet) for sparse visual processing. We begin by presenting a big picture of the neuroscience of visual perception and its optimization for sparse processing. The grounds for our proposition come next. For this, (1) we first review^1^ by reproducing its main results, via a slightly different approach, along also with improving them. In this framework, we show, again, that Gabor-like sets of basis functions, which are similar to the receptive fields of simple cells in V1, are excellent candidates for sparse representation of natural images; i.e., images from the natural world, affirming the brain to have been optimized for this. (2) We then prove that the intra-layer synaptic weight matrices of this region can be reasonably first-order approximated by an identity mapping, and are thus sparse themselves. (3) Finally, we point out that intra-layer weight matrices having identity mapping as their initial approximation, irrespective of this approximation being also a reasonable first-order one or not, resemble the building blocks of direct-shortcut digital ResNets, which completes the grounds. This biological ResNet interconnects the sparsity of the final representation of the image to that of its intra-layer weights.

Further exploration of this ResNet, and understanding the joint effects of its architecture and learning rules, e.g. on its inductive bias^2^, could lead to major advancements in the area of bio-inspired digital ResNets. One immediate line of research in this context, for instance, is to study the impact of forcing the direct-shortcuts to be good first-order approximations of each building block. For this, along with the ${{\ell}}_{1}$-minimization posed on the basis function coefficients the ResNet finally provides at its output, another parallel one could e.g. also be posed on the weights of its residual layers.

## The neuroscience of visual perception

Visual perception begins with the reception of visual stimuli by the retina, where light-sensitive cells convert light into electrical signals. These signals are then transmitted via the optic nerve to the brain's visual processing centers, primarily V1, located in the occipital lobe. Visual information is processed hierarchically, moving from lower-level visual areas to higher-level association ones. This hierarchical organization allows for the extraction of increasingly complex features as the information progresses forward. At the early stages, neurons in V1 respond to simple features such as edges, orientation, and contrast. As information travels along the visual pathway, neurons in higher areas become sensitive to more complex features, including shapes, textures, and object parts.

Core object recognition relies on achieving both invariance and selectivity^3^. Invariance refers to the ability to recognize objects despite variations in their appearance, while selectivity involves responding specifically to certain object features. The ventral visual stream, also known as the “what pathway”, has a crucial role in these^3^. This pathway extends from V1 to the inferotemporal (IT) cortex, passing through intermediate visual areas such as V2 and V4. The IT cortex is particularly important for higher-level object processing, and is involved in representing complex features via subspace untangling^3^. These representations facilitate rapid and accurate recognition.

Visual perception involves a combination of feedforward and feedback processing. Feedforward connections transmit information from lower to higher visual areas, while feedback ones convey contextual information and top-down influences from higher to lower regions. This feedback mechanism helps refine object representations, and contributes to the brain's ability to recognize objects under varying conditions.

The brain's ability in visual perception is shaped by experience and learning. Through exposure to a variety of objects in different contexts, it refines its representation of object features, and develops robust recognition abilities. This process, referred to as plasticity, allows for adaptation to new stimuli and environments.

### Sparse visual processing

Visual perception is also largely founded on sparse processing. This simplifies storage and retrieval of information by the memory^4^, and improves energy efficiency^4^, filtering out noise, memory interference reduction, linear separability of stimuli, and learnability.

Biological sparse visual processing starts in the retina and the lateral geniculate nucleus (LGN) of the thalamus, where forms of contrast normalization, as a type of such processing, are performed. In the next stage, V1 contains neurons that are selective to specific features of visual input, such as orientation and spatial frequency. The receptive fields of these neurons, to be studied thoroughly in the next section, can be modeled using Gabor filters, which are optimal for sparse coding.

Color blobs have also a role in this context. These are clusters of neurons in V1 that are selective to color, and are interspersed with the orientation selective neurons. They are thought to play a role in color constancy, which is the ability to perceive colors accurately under different lighting conditions.

Other regions of the visual cortex such as V2 and V4 have roles as well. V2 contains neurons that are selective to more complex features of the visual input such as curvature and texture. These neurons can be modeled using basis functions more complex than Gabor filters. V4 contains neurons that are selective to more abstract features such as object categories. These are modeled by even more complex basis functions.

#### The roles of the different mechanisms of synaptic plasticity

The major mechanisms of synaptic plasticity and their roles in sparse visual processing are:

1. Long-term potentiation (LTP): This is a form of plasticity that involves a long-lasting increase in the strength of synaptic connections between neurons. It can lead to an increase in the strength of connections between similar neurons, which, as will be scrutinized later, is a key ingredient in shaping a biological ResNet for sparse processing.
2. Long-term depression (LTD): Being the counterpart of LTP, this involves a long-lasting decrease in the connection strengths, which helps prune unnecessary connections and increase the sparsity of the neural code.
3. Spike-timing dependent plasticity (STDP): Depends on the relative timing of pre- and post-synaptic spikes, and can result in either LTP or LTD. It is thought to have influence on learning and memory processes. Its role in sparse processing is mostly through the LTP and LTD it leads to.
4. Homeostatic plasticity: Maintains the overall activity level of neurons within a functional range. It can be achieved through a variety of mechanisms, such as synaptic scaling, which involves adjusting the strength of all synapses onto a neuron by a common factor. This form of plasticity is thought to have impacts on maintaining the stability of neural networks and preventing runaway excitation or inhibition, two phenomena that can disrupt the sparsity of the neural code.
5. Bienenstock-Cooper-Munro (BCM) plasticity: This is thought to have effects on the development of orientation selectivity in the visual cortex. It is based on the Hebbian learning rule, according to which, synapses between neurons that fire together are strengthened. However, it introduces a threshold mechanism that only lets synapses that are active within a certain range of activity levels to be strengthened. This mechanism can help increase the sparsity of the neural code by ensuring that only a small number of neurons are active at any given time.

## The visual cortex is optimized for sparse processing

This section is actually a review of^1^ by reproducing its main results, via a slightly different approach, along also with improving them.

### Dictionary functions for sparse image representation

A generic image $I[m,n]$ with $N$ pixels can be represented as a linear combination of not necessarily orthogonal basis functions $f_{i} \left[ {m,n} \right]$ as

1 $$I\left[ {m,n} \right] = \mathop \sum \limits_{i = 1}^{N} a_{i} f_{i} \left[ {m,n} \right],$$

were $a_{i}$ s are weighting coefficients, and $m$ and $n$ are numbering variables for the rows and columns of the pixels respectively. For a random image with no priori information about it, these coefficients can have any arbitrary value, and nothing can be forecasted about their properties beforehand. Natural images are however not random. They have a variety of properties which can be leveraged to represent them much more efficiently. Among these properties is their pixel redundancy, which has been used traditionally for their compression in the many well-known algorithms such as Jpeg. These algorithms lead to representations with considerable number of the coefficients being close to zero, which can be practically ignored and thus discarded, resulting in sparse representations. The number of non-zero coefficients in such approximations is much smaller than $N$, and the representation is hence much suitable to be deployed in applications such as compression and classification.

To achieve the maximum possible sparseness, a fundamental step is proper design of the dictionary of the basis functions ${f}_{i}$. One approach to this is via an optimization problem^1^ which aims to maximize the sparseness in an approximate representation of the image, while preserving its original information content as much as possible. A cost function to be minimized for this has been proposed in^1^ as

2 $$C = \mathop \sum \limits_{m,n} \left[ { I\left[ {m,n} \right] - \mathop \sum \limits_{i} a_{i}^{\prime } f_{i} \left[ {m,n} \right]} \right]^{2} + \lambda \mathop \sum \limits_{i} S\left( {\frac{{a_{i}^{\prime } }}{\sigma }} \right).$$

In the above, the first summand is a measure of preservation of information in the representation. The second is the cost of the code being non-sparse. The function $S$ in this term can be any typical neural activation function such as $1-{e}^{-{x}^{2}}$, $\text{log}\left(1+{x}^{2}\right)$, $\left|x\right|$, etc., and $\sigma$ is a scaling constant. For any given image, this summand assigns a cost to the computed code depending on how activity is distributed among the coefficients. Representations in which activity is distributed among more coefficients result in higher costs, and vice versa. $\lambda$ is a positive factor that weighs the importance of the second term relative to the first.

The retina has a log-dynamic transfer function, which, as we have investigated in our experiments, improves the sparseness of the neural code further. To model this, we modify (2) as

3 $$C_{{{\text{log}}}} = \mathop \sum \limits_{m,n} \left[ {\log \left( {I\left[ {m,n} \right]} \right) - \mathop \sum \limits_{i} b_{i}^{\prime } g_{i} \left[ {m,n} \right]} \right]^{2} + \lambda \mathop \sum \limits_{i} S\left( {\frac{{b_{i}^{\prime } }}{\sigma }} \right),$$

where the logarithm is applied element-wise, $g_{i}$s are the basis functions in the new representation $\text{log}(I\left[m,n\right])={\sum }_{i=1}^{N}{b}_{i}{g}_{i}\left[m,n\right]$, and $b_{i}^{\prime }$s are approximations to $b_{i}$s.

For a selected set of basis functions $g_{i}$, a set of $b_{i}^{\prime }$s ($1 \le i \le N$) can be computed using numerical methods such as the conjugate gradient algorithm. A sparse representation of the image would then be given simply by taking the coefficients with the largest absolute value and discarding the rest. For the case of $\lambda = 0$, in which sparsity is considered to be of no importance, $b_{i}^{\prime }$s would be equal to their counterparts $b_{i}$, and the cost function would trivially reduce to 0.

Of course the atoms, which are the most important component of the problem, are not available beforehand. To compute them, a set of suitable or otherwise random ones are usually picked initially, which are then optimized by minimizing (3) iteratively over an ensemble of training images. The whole algorithm has been compactly presented in Table 1. After an enough number of iterations, the cost function will reach a practically constant value, and the final optimized atoms emerge.

**Table 1:**
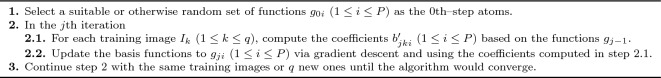
The update algorithm for computing $P$ basis functions via (3). To have a complete basis, we should set $P=N$.

The above algorithm has two notable properties: (1) it poses no a priori condition on the atoms, such as being orthogonal, etc. Also, (2) there are no restrictions on their number. We can choose to design an over-complete set of atoms with more than $N$ members, resulting in sparser, but more computationally intensive, representations. On the other hand, they can be fewer than $N$, leading to a basis which, despite being incomplete, can be derived via lighter computations.

One key consideration about the above procedure is its proper initialization. Selection of a suitable starting point will result in its fast convergence, and prevents it from getting trapped in local minima as well. The starting point we propose here is inspired from the structure of the receptive fields of simple cells in V1, which are (1) spatially localized, (2) oriented, and (3) bandpass. The process is explained next.

### The receptive fields of simple cells in the primary visual cortex as the 0th-estimate atoms

Human's brain has a very efficient performance in perception of natural images. A probable reason is that natural images have sparse structures, a property that reduces their processing load for the brain. It is therefore natural to expect that the receptive fields of simple cells in V1, which are image patterns to which the visual system has its highest sensitivity, would probably be proper candidates for the algorithm's initialization.

**Figure 1:**
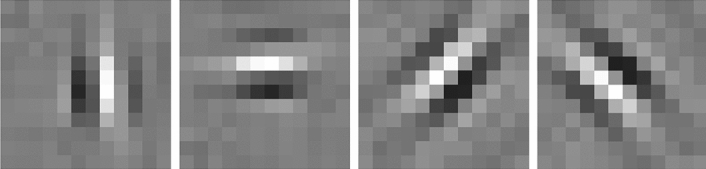
Four samples from the 0th estimate elements of the sparse coding dictionary.

To evaluate this, we have designed a set of atoms having the above three properties of these fields: being spatially localized, oriented, and bandpass, with spatial frequency bandwidth and aspect ratio (length/width) of approximately 1.5 Hz and 2 respectively. The image size we chose for this proof-of-concept experiment is $12\times 12$. To have a slightly over-complete basis set (with $12\times 12+1=145$ atoms), we first designed 72 starting atoms by wavelet filtering of simple 3 to 5-pixels-long lines (edges) at random locations and directed towards the directions of 0 and 45°, along with their 90° rotated counterparts (a total of 144 functions). Four samples from these atoms have been depicted in Fig. 1. While we deployed a Daubechies wavelet for this purpose, the type of the used wavelet does not affect the results considerably. A single extra code with uniform intensity over all its pixels was also added to the set to function as a low-pass component, implementing the mean (average of the pixel intensities) of the image to be coded. We therefore end up with an over-complete 0th estimate sparsity domain.

The above design is of course just a typical one proposed by the author, and can be replaced with many alternatives.

### Experimental results

The final atoms are derived by minimizing (3) with the above kernels as its initializing ${g}_{0i}$s. Experimental results, to be presented next, show that compared to the case where random atoms are used as initializers, this leads to impressive decrements in the number of the required iterations for the algorithm to converge. The finally obtained atoms would have the three properties of being localized, oriented, and bandpass as well. However, one may choose not to perform the optimization at all, and deploy the draft atoms directly as the final dictionary. Simulations show that these drafts, although being suboptimal, are also adequate approximations resulting in acceptable sparseness.

We test the performance of our designed codes in the above two separate scenarios of being the initializers, as well as their direct deployment as the final dictionary. The tests are performed on ten $540\times 540$ images of natural scenes which have been segmented to $12\times 12$ frames, the latter being the code size. We first pre-process these segments by passing them through the zero-phase whitening/low-pass filter

4 $$H\left( {{\text{e}}^{j\omega } } \right) = \frac{\omega }{2\pi }{\text{e}}^{{ - \;\left( {\frac{\omega }{0.4\pi }} \right)^{4} }} .$$

The Whitening implemented by $\frac{\omega }{2\pi }$ compensates for the drawback that the mean square error computed in (3) performs in favor of the low frequency components of the image. The exponential term attenuates the high frequency artefacts produced by rectangular sampling. The results are explained below.

#### Deployment as initializers

In this test we compare the (1) convergence rate, (2) sparsity ratio, and (3) reconstruction error with the case where random functions are used as initializers. The atoms are updated every 100 image segments, and are scaled at each update to ensure equal variance for each coefficient over the process. The function $S$ is $\text{log}\left(1+{x}^{2}\right)$, the factor $\sigma$ equal to the pixel variance of the filtered images, and the weighing parameter is set as $\lambda =0.15\sigma$. We stop when the variance of the cost function $C$ gets equal to 1% of its mean.

1. For the case of random initialization, the algorithm converges after 3000 iterations (300k image segments). When initialized with the proposed atoms, this number reduces to 600, corresponding to a remarkable 80% reduction.
2. The sparsity ratios in the two cases are almost identical: a reconstructed image having a 97% correlation with the original one emerges in the first case using the 43 largest coefficients on average. In the 2nd, such an image emerges using the slightly smaller number of 41 ones.
3. The average reconstruction errors are equal respectively to 5.7% and 5.2% of the pixel variance of the original image. This confirms that the reduced computational load of our proposed initialization does not affect the mean square error (MSE) of the reconstruction adversely.

#### Direct deployment as the final dictionary

The proposed atoms have also been used in another test directly as the final dictionary, without any further optimization. The average MSE of the sparsely represented images here is 9.4% of their pixel variances, which is a rather good result for such a simple design. Also, a reconstruction having a 97% correlation with the original counterpart can be realized here using the 56 largest coefficients on the average, demonstrating a rather acceptable performance in terms of sparsification as well.

## The intra-layer weight matrices in V1 can be first-order approximated by an identity mapping component

Synaptic strengths (weights) in V1 have been experimentally shown in^5^ to be strong between neurons with the most correlated responses, and weak otherwise. The mathematical reason of this, which has its roots in the statistical dynamics of the firing of the associated neurons, has not however been presented there. This reason is nonetheless a fundamental subject, and can be a first question. In this section we first show that this synaptic organization can be derived mathematically from the Hebbian learning dynamics that govern the brain's process of learning. We then highlight its implication on the structure of the corresponding intra-layer synaptic weight matrices.

### Proposition

Synaptic strengths in V1 are strong between neurons with the most correlated responses, and weak otherwise^5^.

### Proof

Hebbian learning is one of the dominant learning mechanisms governing the brain’s neuronal networks. Denoting the weight between the pre- and post-synaptic neurons ${n}_{i}$ and ${n}_{j}$ by ${w}_{ij}$, the firing rate of any neuron $n_{k}$ by $f_{k}$, and its mean by $\bar{f_k}$, we propose the Hebbian rule

5 $$\frac{{\text{d}}}{{{\text{d}}t}}w_{ij} \left( t \right) = c_{1} \bar{f_{i}}^{\alpha } w_{ij}^{\beta } \left( t \right)\bar{f_{j}}^{\gamma } - c_{2} w_{ij} \left( t \right)$$

for the neurons in V1, where $t$ denotes the time. In the above, the first and second terms on the right side implement the corresponding Hebbian growths and synaptic decays respectively, and ${c}_{1}$, ${c}_{2}$, $\alpha$, $\beta$, and $\gamma$ are adjustable parameters. For the weights to converge, as well as for preventing them from being subjugated by only a few, these parameters have of course to fulfill some regularity conditions, to be discussed shortly. At the steady state, the left and thus the right side of the equation will be equal to 0, and the weights are therefore derived as

6 $$w_{ij} = c\bar{f_{i}}^{{\frac{\alpha }{1 - \beta }}} \bar{f_{j}}^{{\frac{\gamma }{1 - \beta }}} \quad ({c = \left( {\frac{{c_{1} }}{{c_{2} }}} \right)^{{\frac{1}{1 - \beta }}} }).$$

It is well-known^6^ that neuronal firing rates and their means have log-normal distributions. Because the sum of normal random variables is also normal, the powers and product of log-normal variables are also log-normal. As scaled powers of such variables, for any $j$, $w_{ij}$s are therefore also as such, as also figured out in^6^ experimentally.

Assuming a linear post-synaptic activation function in the unsaturated regime, we also have $f_j=\sum_i w_{ij}f_i$. Considering the summands on the right side of this being log-normal (they are products of such variables), their sum ($f_j$) being also as such can happen only if they are dominated by just a few, and are also highly correlated. Otherwise, based on the central limit theorem, they would sum up to a normal distribution. The mentioned correlations naturally imply the similarity of the dominating neurons with $n_j$ as well, which completes the proof. ■

In matrix terms, the above organization of synaptic strengths corresponds to an intra-layer weight matrix that can be first-order approximated by an identity mapping component.

The regularity conditions referred to in the above prevents the dominating summands from getting too few, allowing for the existence of lots of healthy and active, although small and residual, neural connections. Without them, many of the connections may become actually ill or broken.

Equation (5) also explains how correlated firing rates strengthen the synaptic weights of neurons with similar responses (and, in fact, functions), and weaken them otherwise. Strong correlations increase the first term on the right, and thus left side of (5), meaning a higher rate of increase for the synaptic weight, and vice versa. This is in fact the algorithmic origin of the functional organization of neurons, proved in^5^ only experimentally.

## Intra-layer weight matrices having a first-order identity mapping approximation resemble the building blocks of direct-shortcut digital ResNets

### Deep residual learning

A widely adopted approach to enhance the performance of neural networks is to increase the number of their layers, the result being referred to as deep neural networks. Deeper nets are however more difficult to train. They; e.g., converge slowly. Deep residual learning^7^, which was first released in 2015 and then presented in CVPR 2016, is a framework to ease the training of networks that are substantially deeper than those used previously.

**Figure 2:**
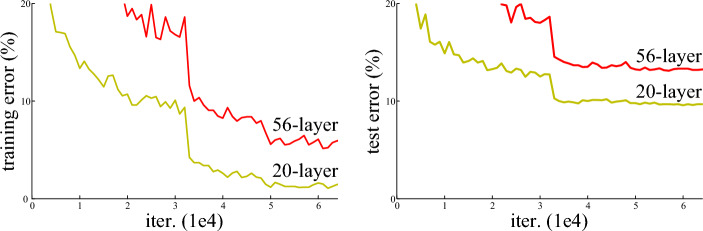
Training error (left) and test error (right) on CIFAR-10 with 20-layer and 56-layer “plain” networks. The deeper network has higher training error, and thus test error (source:^7^).

When deep nets start to converge, a degradation problem arises: as the network depth increases, accuracy gets saturated, and then degrades rapidly. Unexpectedly, this degradation is not caused by over-fitting, and adding more layers even to a suitably deep model increases even the training error (Fig. 2). While the reason is not clear yet, it is conjectured in^7^ to be the probable exponentially-low convergence rates of deep plain nets.

This phenomenon indicates that not all systems are similarly easy to optimize. One conceptual workaround on this, referred to as deepening “by construction”^7^, is to create a deeper net by adding identity mapping layers to a shallower one. In theory, the existence of this solution indicates that a deeper net should produce errors no higher than that of the shallower one. But experiments show that plain nets are generally not able to find solutions comparable to this handcrafted deeper net, at least in a feasible time.

**Figure 3:**
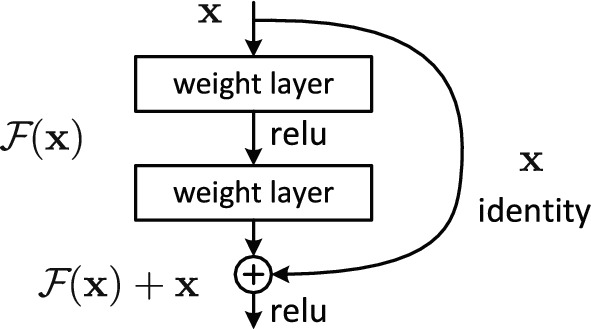
Residual learning: a building block (source:^7^).

**Figure 4:**
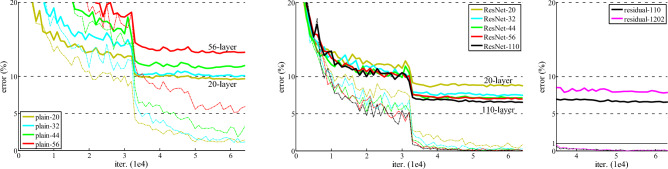
Training on CIFAR-10. Dashed and bold lines denote training and testing errors respectively. **Left**: plain networks. The error of plain-110 is higher than 60% and not displayed. **Middle**: ResNets. **Right**: ResNets with 110 and 1202 layers (source:^7^).

The idea of ResNets addresses this drawback. Let $\mathcal{H}({\mathbf{x}}\text{)}$ be an underlying mapping to be fit by a few stacked layers, with ${\mathbf{x}}$ denoting the input to their first. If one hypothesizes that multiple nonlinear layers can asymptotically approximate any complicated function such as $\mathcal{H}(\mathbf{x})$ (this hypothesis is of course still an open question), and assuming that the input and output are of the same dimensions, they could asymptotically approximate the residual function ${\mathcal{F}}({\mathbf{x}}{)}: = {\mathcal{H}}({\mathbf{x}}{)} - {\mathbf{x}}$ as well. ResNets actually let the stacked nonlinear layers fit ${\mathcal{F}}$. The original mapping is then recast into ${\mathcal{F}}({\mathbf{x}}{)}\;{ + }\;{\mathbf{x}}$, a formulation that can be realized by feedforward neural networks with shortcut connections (Fig. 3). The justification for this^7^ is that if an identity mapping was optimal, it would be easier to push the residual to zero rather than to fit the original mapping by a stack of nonlinear layers. This results in a faster convergence, along also with a lower final error (Fig. 4), and partially compensates for the convergence drawback of deepness.

### Direct shortcuts

**Figure 5:**
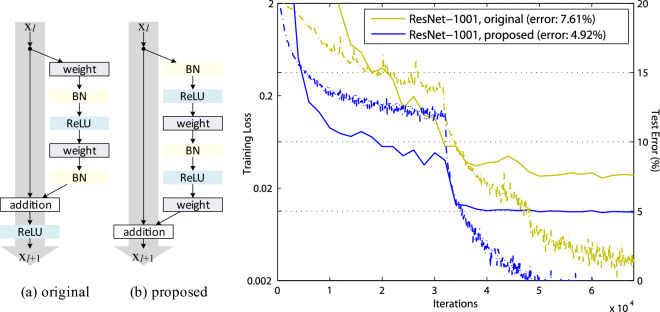
**Left**: (a) original residual unit in^7^, (b) direct residual unit^8^. The gray arrows indicate the easiest paths for the information to propagate, corresponding to the additive component $\mathbf{x}$ in $\mathcal{H}(\mathbf{x})$. **Right**: training curves of 1001-layer indirect- and direct-shortcut ResNets on CIFAR-10. Solid lines denote test error (y-axis on the right), and dashed lines denote training loss (y-axis on the left). The direct unit, as also expected from theory, makes ResNet-1001 easier to train (source^8^).

The authors of^7^ have analyzed the propagation of signals within the residual building blocks in^8^. The results suggest that for an even easier training, forward and backward signals could directly propagate not only within a residual unit, but across the entire network, through direct identity mapping components (shortcuts) that skip after-addition activations (Fig. 5). Motivated by this, they propose a new residual unit ((b) on the left side of Fig. 5), which makes training easier and improves generalization. They also perform a series of experiments that support the impact of these direct shortcuts (Fig. 5, right).

#### Resemblance by the intra-layer weight matrices of V1

Considering the above, intra-layer weight matrices having identity mapping as their initial approximation, irrespective of it being also a good first-order one or not, resemble the building blocks of direct-shortcut digital ResNets. This, specifically, is the case about the intra-layer weight matrices of V1.

## Conclusion

Let us now wrap up what we studied. We first observed that V1 is optimized for sparse processing. We then proved that the intra-layer synaptic weight matrices of this region can be reasonably first-order approximated by identity mappings, and are thus sparse themselves. Finally, we showed that intra-layer weight matrices having identity mapping as their initial approximation, irrespective of it being also a reasonable first-order one or not, resemble the building blocks of direct-shortcut digital ResNets. We then proposed, based on these three grounds, that the primary visual cortex could be thought of as a biological direct-shortcut ResNet for sparse visual processing.

The implications of identity mappings being good first-order approximations for the layers of this biological ResNet on bio-inspired digital ResNets are among the many topics around this proposition to be explored. To implement this property digitally, along with the ${{\ell}}_{1}$-minimization posed on the basis function coefficients the ResNet finally provides at its output, another parallel one could e.g. also be posed on the weights of its residual layers.

Besides the intra-layer skip connections in V1 studied here, the brain also benefits from inter-area such connections, referred to as skip pathways. These are neural circuits that provide alternative routes for signal transmission, and are crucial for maintaining functionality when primary pathways are damaged or disrupted. In the neocortex; e.g., cortical V4 neurons that get inputs from V1 skip the intermediate layers. Skip connections contribute to the brain's resilience and neuroplasticity, allowing it to adapt and reorganize itself.

## Data Availability

Image datasets available at https://web.sas.upenn.edu/upennidb.
